# Impact of sequence selection on postoperative MRI interpretability and artifact reduction in simulated mandibular trauma

**DOI:** 10.3389/froh.2026.1778789

**Published:** 2026-07-31

**Authors:** Daniel Zedler, Adib Al-Haj Husain, Sameena Sandhu, Maximilian Eberhard Hermann Wagner, Bernd Stadlinger, Harald Essig, Yesenia Gassner, Thomas Frauenfelder, Egon Burian

**Affiliations:** 1 Department of Diagnostic and Interventional Radiology, University Hospital Zurich, University of Zurich, Zurich, Switzerland; 2 Department of Cranio-Maxillofacial and Oral Surgery, University Hospital Zurich, University of Zurich, Zurich, Switzerland; 3 Department of Cranio-Maxillofacial Surgery, GROW School for Oncology and Reproduction, Maastricht University Medical Centre, Maastricht, Netherlands; 4 Clinic of Cranio-Maxillofacial and Oral Surgery, Center of Dental Medicine, University of Zurich, Zurich, Switzerland

**Keywords:** artifacts, diagnostic imaging, magnetic resonance imaging, mandibular fractures, osteosynthesis

## Abstract

**Introduction:**

This *ex vivo* study evaluated the diagnostic performance of different MRI sequences for postoperative imaging of simulated mandibular fractures stabilized with titanium and bioresorbable osteosynthesis materials.

**Methods:**

Six porcine mandibles with standardized fractures of the mandibular angle, body, and parasymphysis were stabilized using titanium microplates, titanium reconstruction plates, or bioresorbable systems. All specimens underwent photon-counting computed tomography (CT) as a reference and 3 Tesla MRI, including five sequences: T1-weighted turbo spin echo (T1-TSE), DIXON-based imaging (DIXON), T1-TSE with vendor-specific artifact reduction techniques (WARP), ultrashort echo time (UTE), and slice encoding for metal artifact correction (SEMAC). Three blinded observers rated general image interpretability, fracture visibility, artifact extent, and soft- as well as hard-tissue interpretability using five-point Likert scales. Quantitative analysis included signal-to-noise ratio (SNR), contrast-to-noise ratio (CNR), and two-dimensional artifact measurements.

**Results:**

Advanced artifact-reduction sequences outperformed conventional T1-TSE and DIXON (*p* < 0.001). UTE and WARP achieved the highest general image interpretability (median 5) and excellent fracture line visibility, alongside SEMAC. WARP provided the best soft-tissue interpretability (median 5), highest muscle SNR (median 207.9 vs. 74.4 for SEMAC and 51.9 for UTE; *p* ≤ 0.013), and highest CNR (median 184.8 vs. 59.6 for SEMAC and 23.5 for UTE; *p* ≤ 0.043), while UTE demonstrated superior hard-tissue SNR (median 30.0 vs. 13.2 for SEMAC; *p* < 0.001). SEMAC showed the greatest reduction in artifact extent (135% relative to CT vs. 209% for WARP and 220% for UTE; *p* ≤ 0.004). There was good inter-reader agreement (*α* = 0.76).

**Conclusion:**

Advanced MRI sequences, such as SEMAC, UTE and WARP, substantially improved postoperative image interpretability and reduced implant-related artifacts. A tailored MRI protocol may complement CT in selected postoperative scenarios.

## 1 Introduction

Mandibular fractures are among the most common fractures of the facial skeleton and require comprehensive clinical and radiological evaluation to enable multidisciplinary coordinated state-of-the-art treatment (1–3). Multidetector computed tomography (MDCT) and cone-beam computed tomography (CBCT) are currently the gold standard due to their excellent anatomical detail, rapid acquisition, and high diagnostic sensitivity (4, 5).

With recent advancements in dentomaxillofacial imaging over the past decade, magnetic resonance imaging (MRI) has expanded to a wider range of clinical applications. MRI offers complementary advantages, particularly for assessing soft-tissue pathology, temporomandibular joint (TMJ) involvement, nerve integrity, and inflammatory disease without radiation exposure, which is of pivotal importance in young patients (6–11). Recent developments in CT-like MRI techniques, including ultrashort echo time (UTE) and advanced gradient-echo–based approaches, have expanded the role of MRI toward improved bone visualization (12–14). Despite these advances, postoperative mandibular imaging with MRI remains challenging due to susceptibility artifacts caused by osteosynthesis materials and implants, which can obscure adjacent bone and soft tissues and substantially limit diagnostic interpretability (15, 16).

Several artifact-reduction techniques have been introduced to address metal-related distortions, including slice encoding for metal artifact correction (SEMAC), view-angle tilting (VAT), which can be incorporated into dedicated artifact reduction techniques such as WARP (vendor-specific sequence by Siemens Healthineers, Forchheim, Germany), and ultrashort echo time (UTE) imaging (17–19). These techniques have demonstrated artifact reduction in various anatomical regions. Their performance has been evaluated by Rendenbach et al. in an *in vitro* setting but not in postoperative mandibular imaging — particularly regarding diagnostic interpretability of both osteosynthesis material and fracture line delineation (16).

Specifically, it remains unclear which advanced MRI sequences provide the most favorable balance between artifact suppression, fracture line visibility, and tissue interpretability in the presence of commonly used mandibular osteosynthesis materials. Controlled experimental data comparing these techniques under standardized conditions are lacking. Therefore, this *ex vivo* study aimed to systematically compare conventional and advanced MRI sequences for postoperative mandibular fracture imaging using a porcine fracture model stabilized with titanium and bioresorbable osteosynthesis systems. We hypothesized that advanced artifact-reduction sequences (SEMAC, WARP, and UTE) would provide superior diagnostic interpretability and reduced artifact extent compared to conventional T1-weighted TSE and DIXON imaging.

## 2 Material and methods

### 2.1 Study design and ethics

Porcine mandibles were selected because their anatomical and morphological characteristics closely resemble those of the human craniofacial skeleton. Moreover, their reliability and frequent application in orofacial research have been well documented (20). The study was conducted in accordance with ethical guidelines approved by the Office of Animal Welfare and 3R at the University of Zurich. Reporting of the experimental procedures followed the ARRIVE (Animal Research: Reporting of *in vivo* Experiments) standards where applicable, adapted for *ex vivo* study design. According to local regulations and confirmation by the Office of Animal Welfare and 3R at the University of Zurich, no formal animal experimentation approval was required for the use of slaughterhouse-derived specimens.

### 2.2 Specimen preparation

A total of six young porcine mandibles were obtained from a local slaughterhouse in Zurich, Switzerland. The surgical procedures were performed by three clinicians: M.E.H.W., a board-certified oral and maxillofacial surgeon with 17 years of experience; S.S., also board-certified with 13 years of experience; and A.A.H., a resident with four years of training. Surrounding soft tissues were carefully dissected and subsequently repositioned and sutured to preserve anatomically realistic conditions within the *ex vivo* model. The standardized mandibles were each assigned six fractures at clinically relevant sites (angle, body, and parasymphysis bilaterally), totaling 36 fractures. Fixation was accomplished using different osteosynthesis systems, including titanium microplates of varying thicknesses (0.6 mm, 0.8 mm, and 1.0 mm), each secured with maxDrive titanium screws (1.5 mm diameter, lengths of 5 mm or 6 mm; KLS Martin Group, Tuttlingen, Germany). Additionally, straight titanium MatrixMANDIBLE reconstruction plates with thicknesses of 2.0 mm, 2.5 mm, and 2.8 mm were employed and fixed using MatrixMANDIBLE screws (2.4 mm diameter, 8 mm length; KLS Martin Group, Tuttlingen, Germany). A bioresorbable Delta 1.7 copolymer plate (0.75 mm thick) was also utilized, stabilized with Delta screws (1.7 mm diameter, 4 mm length; Stryker, Kalamazoo, MI, USA). The assignment of osteosynthesis devices to the mandibles and fracture sites was randomized, ensuring balanced distribution of each plate type across the different anatomical locations.

### 2.3 Imaging data acquisition

Trained clinical staff and research personnel from the Departments of Radiology as well as Cranio-Maxillofacial and Oral Surgery at the University Hospital Zurich conducted the CT and MRI scans on each porcine mandible. The imaging protocols involved the reference standard photon-counting CT imaging with 130 keV (NAEOTOM Alpha, Siemens Healthineers, Forchheim, Germany), following established clinical protocols. Additionally, MRI examinations were performed on each subject using a 3 Tesla (T) MRI system (MAGNETOM Vida fit, Siemens Healthineers, Forchheim, Germany) with a dedicated 20-channel head and neck coil (Siemens Healthineers, Forchheim, Germany). A five sequence MRI protocol, optimized in-house specifically for trauma assessment, was acquired. These consisted of: (1) an isotropic, prototype ultrashort echo time (UTE) sequence, (2) a slice encoding for metal artifact correction (SEMAC) sequence, (3) a prototype metal artifact reduction package (WARP, vendor-specific artifact reduction techniques) sequence, combining T1-TSE and view-angle tilting (VAT), (4) DIXON sequences and (5) a standard T1-weighted turbo spin echo (T1-TSE) sequence. Acquisition parameters are shown in Table 1.

**Table 1:** Acquisition parameters of MRI sequences.

Sequence	Repetition time (ms)	Echo time (ms)	Flip angle (degrees)	Bandwidth (Hz/Px)	Fat suppression	Matrix read/phase	Voxel size (mm)	Acquisition time (min)	Acceleration factor
UTE	4.6	0.04	5	1,184	None	384 × 384	0.6 × 0.6 × 0.6	3:05	1
SEMAC	4,130	47	120	599	None	384 × 384	0.6 × 0.6 × 2.5	5:55	8
WARP	700	6.9	131/90	507	None	704 × 704	0.3 × 0.3 × 3.0	1:16	4
DIXON	3,400	32	134	446	(*)	336 × 448	0.5 × 0.5 × 2.5	2:48	2
T1	810	15	150	201	None	672 × 380	0.25 × 0.25 × 2.5	2:24	2

Acquisition parameters of the five MRI sequences used, including repetition time (TR) in milliseconds (ms), echo time (TE) in milliseconds, flip angle in degrees, receiver bandwidth in hertz per pixel (Hz/Px), whether fat suppression methods were used, matrix in read and phase direction, voxel size in millimeters (mm), total acquisition time in minutes (min) and acceleration factor. For DIXON acquisitions, water-only images (i.e., representing fat-suppressed images) were reconstructed (*).

UTE, ultrashort echo time; SEMAC, slice encoding for metal artifact correction; WARP, vendor-specific artifact reduction techniques (Siemens Healthineers, Forchheim, Germany); DIXON, DIXON-based imaging; T1, T1-weighted turbo spin echo; TR, repetition time, TE, echo time.

### 2.4 Image analysis

CT and MRI datasets were saved in DICOM format and assessed using the local Picture Archiving and Communication System (DeepUnity Diagnost 2.0.2.2, Dedalus, Florence, Italy). MRI sequences were evaluated by three observers with different levels of expertise and specialization: D.Z., a resident in radiology with one year of experience; A.A.H., a resident in oral and maxillofacial surgery with four years of experience; and E.B., a board-certified radiologist with 10 years of experience.

### 2.5 Qualitative assessment

Three independent readers (radiology resident, oral and maxillofacial surgery resident, board-certified radiologist) assessed the MRI datasets. Readers were blinded to implant type but not to sequence type, as sequence characteristics are inherently distinguishable. Image quality was assessed for: (1) general image interpretability, (2) visibility of the osseous fracture line, (3) artifact extent, (4) diagnostic interpretability of soft tissues, and (5) diagnostic interpretability of hard tissues. These were analyzed using a five-point Likert scale established for oral and maxillofacial trauma evaluation in MRI: 5 = excellent, no restrictions for clinical use, (almost) absent artifacts; 4 = very good, containing no substantial adverse effect for clinical use, minimal artifacts; 3 = average, borderline clinical use due to the image quality, moderate artifacts; 2 = poor, substantial adverse effect for clinical use, severe artifacts; and 1 = very poor, not suitable for clinical use, extensive artifacts (21).

### 2.6 Quantitative assessment

**Figure 2:**
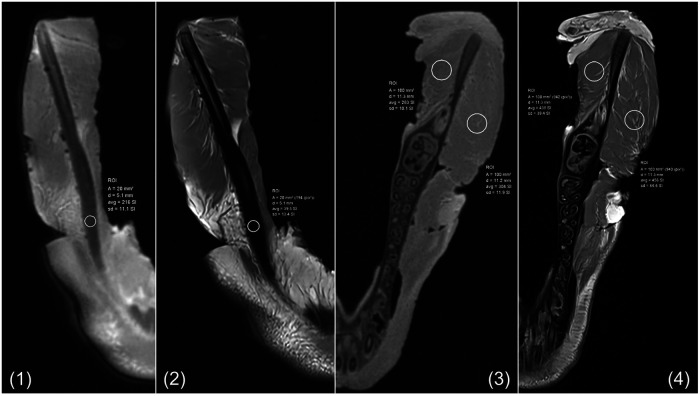
Signal intensity measurements in hard and soft tissues. Examples of signal intensity measurements in the hard tissues/cortical bone (1–2) and soft tissues/muscle (3–4). Sample images represent UTE (1 and 3) and WARP (2 and 4). From left to right: (1) Signal intensity measured in cortical bone (mandibular body) in UTE sequence, (2) signal intensity measured in cortical bone in WARP sequence, (3) signal intensity measured in the muscles (M. pterygoideus medialis and M. masseter) in UTE, (4) signal intensity measured in the muscles in WARP. Signal intensity values were then used to determine background-noise-based signal-to-noise ratio (SNR) and contrast-to-noise ratio (CNR). UTE, ultrashort echo time; WARP, vendor-specific artifact reduction techniques (Siemens Healthineers, Forchheim, Germany); SNR, signal-to-noise ratio; CNR, contrast-to-noise ratio.

To support our qualitative assessment of diagnostic interpretability of the soft and hard tissues, background-noise-based SNR analysis was carried out in the advanced MRI sequences (UTE, SEMAC, and WARP) in the hard tissue (cortical bone) as well as the soft tissue (muscle, see Figure 2). Background-noise-based SNR was calculated as follows: $\mathrm{SNR}=\;\frac{\mu (\mathrm{ROI})}{\sigma (\mathrm{air})}\;$, where *μ*(ROI) corresponds to the mean signal in the tissue of interest (cortical bone or muscle) and *σ*(air) corresponds to the standard deviation in the air/background (22). SNR values were not corrected for voxel size or other sequence parameters as all sequences were evaluated in an *ex vivo* porcine mandible model to reflect their clinical performance. Additionally, contrast-to-noise ratio (CNR) was calculated as the absolute difference between the mean signal intensities of hard tissue (μ(cortical bone)) and soft tissue (μ(muscle)) divided by the standard deviation of image noise in the air/background (*σ*(air)): $\mathrm{CNR}\;=\;\frac{\left[\mu (\mathrm{muscle})-\mu (\mathrm{bone})\right]}{\sigma (\mathrm{air})}$. In the presence of parallel imaging, advanced artifact-reduction techniques, and non-linear reconstruction, these conventional SNR and CNR values represent relative rather than absolute measures of image quality.

Metal-related artifact extent was additionally quantified by measuring the two-dimensional artifact area adjacent to osteosynthesis material on MRI in axial plane, measuring length and width and calculating area by assuming a rectangular shape. Artifact area was then compared with the corresponding anatomical region on CT. This quantitative approach should be regarded as exploratory, as MRI artifacts can be defined and measured using different methodological approaches, and a rectangular assumption may not fully reflect the complex artifact geometry.

### 2.7 Statistical analysis

Statistical analysis was conducted using SPSS (version 31.0.0.0, IBM Corp., Armonk, NY, USA, 2024). Qualitative data are reported as medians and interquartile ranges and compared using the Friedman test. Interobserver agreement was assessed using Krippendorff's alpha.

Quantitative variables were tested for normality using the Shapiro–Wilk test. Non-parametric tests (Friedman test and pairwise *post hoc* comparisons with Bonferroni adjustment) were performed as appropriate. A two-sided *p*-value < 0.05 was considered statistically significant.

## 3 Results

### 3.1 Qualitative assessment

#### 3.1.1 General image interpretability, fracture delineation and artifact extent

General image interpretability was ranked highest for UTE and WARP (both median 5, IQR 5-5), outperforming SEMAC and DIXON (both median 4, IQR 4-5) as well as T1 (median 4, IQR 4-4). UTE, SEMAC, and WARP showed excellent osseous fracture line visibility (all median 5, IQR 5-5), whilst both DIXON (median 4, IQR 4-5) and T1 (median 3, IQR 2-3) were inferior regarding the delineation of the osseous fracture.

SEMAC showed a superior performance in the evaluation of artifact extent (median 4, IQR 3-5), compared to UTE, WARP, and DIXON (all median 3, IQR 3-4) as well as T1 (median 3, IQR 2-3).

#### 3.1.2 Diagnostic interpretability of soft tissue and hard tissue

Best diagnostic interpretability of soft tissues was achieved with WARP (median 5, IQR 5-5), indicating excellent visualization. UTE and SEMAC (both median 4, IQR 4-4) showed minor limitations in soft tissue assessment, similar to DIXON and T1 sequences (both median 4, IQR 4-5). Both UTE and WARP showed the best diagnostic interpretability of hard tissues (both median 5, IQR 5-5), demonstrating optimal depiction. All other sequences were ranked lower (all median 4, IQR 4-4), reflecting a slight impairment of diagnostic interpretability.

#### 3.1.3 Statistic analysis and interobserver agreement

Friedman test revealed significant differences between all groups (*p* < 0.001). Interobserver agreement using Krippendorff's alpha showed good reliability (*a* = 0.76).

**Figure 1:**
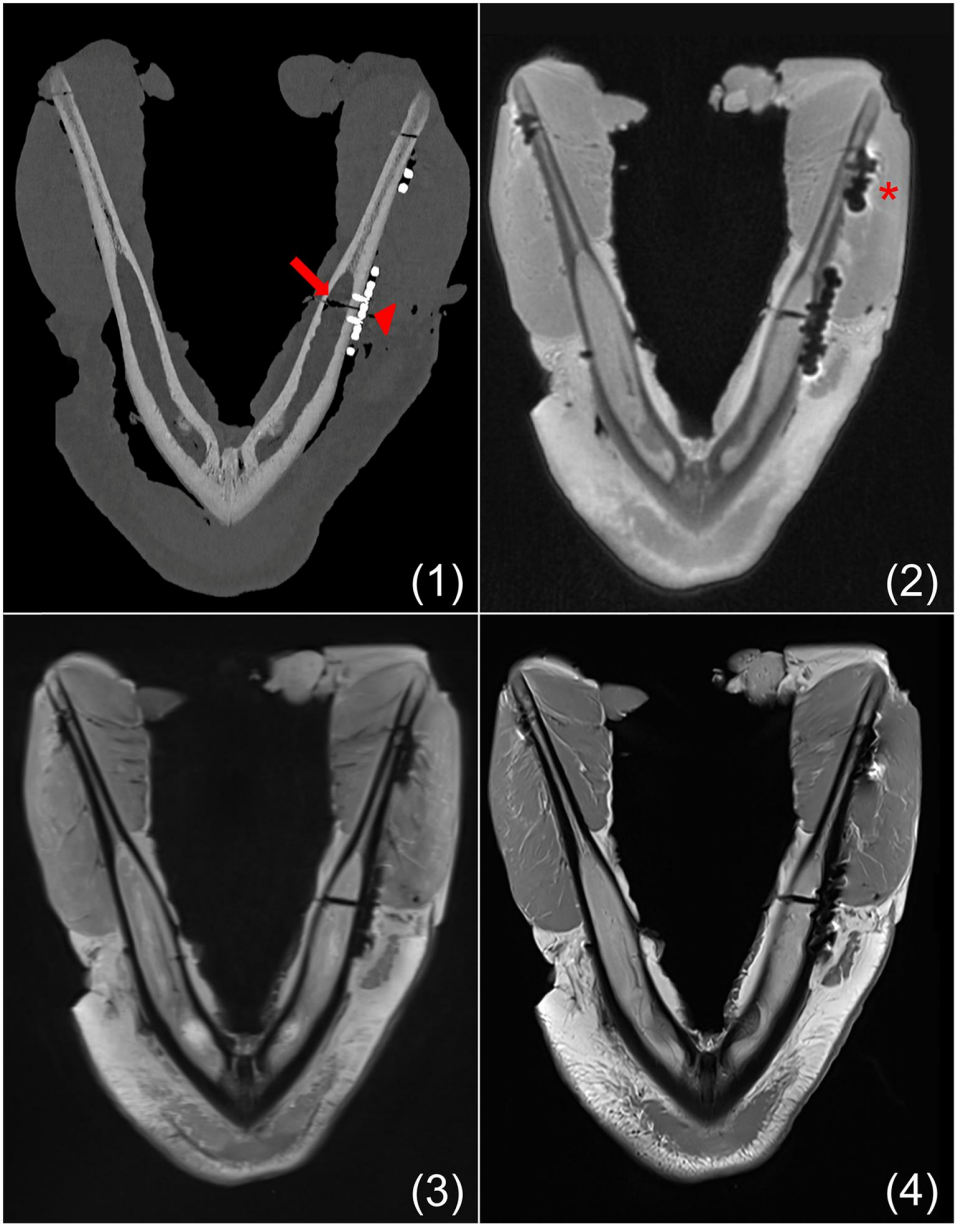
CT versus metal artifact-reduced MRI. Axial imaging of the mandible after osteosynthesis: CT (1) vs. MRI (2–4) with advanced metal artifact-reduction sequences. From left to right and from top to bottom: (1) Photon-counting CT, (2) UTE, (3) SEMAC and (4) WARP. A body fracture is depicted on the left side of the mandible (red arrow in 1) with an adjacent plate osteosynthesis (red arrowhead in 1). Osteosynthesis material and corresponding metallic artifacts are also visible at the mandibular angle (red asterisk in 2). CT, computed tomography; MRI, magnetic resonance imaging; UTE, ultrashort echo time; SEMAC, slice encoding for metal artifact correction; WARP, vendor-specific artifact reduction techniques (Siemens Healthineers, Forchheim, Germany).

For detailed results, see Table 2. Example images are shown in Figure 1.

**Table 2:** Qualitative MRI sequence assessment.

Sequence	General image interpretability, median (IQR)	Visibility of osseous fracture line, median (IQR)	Artifact extent, median (IQR)	Diagnostic interpretability of soft tissues, median (IQR)	Diagnostic interpretability of hard tissues, median (IQR)
UTE	5 (5–5)	5 (5–5)	3 (3–4)	4 (4–4)	5 (5–5)
SEMAC	4 (4–5)	5 (5–5)	4 (3–5)	4 (4–4)	4 (4–4)
WARP	5 (5–5)	5 (5–5)	3 (3–4)	5 (5–5)	5 (5–5)
DIXON	4 (4–5)	4 (4–5)	3 (3–4)	4 (4–5)	4 (4–4)
T1	4 (4–4)	3 (2–3)	3 (2–3)	4 (4–5)	4 (4–4)

Qualitative assessment of the five MRI sequences using a five-point Likert scale.

UTE, ultrashort echo time; SEMAC, slice encoding for metal artifact correction; WARP, vendor-specific artifact reduction techniques (Siemens Healthineers, Forchheim, Germany); DIXON, DIXON-based imaging; T1, T1-weighted turbo spin echo.

### 3.2 Quantitative assessment

#### 3.2.1 SNR/CNR measurements and artifact quantification

SNR analysis revealed that UTE and WARP had significantly higher SNR values than SEMAC in hard tissue (median 30.0 and 23.8, respectively, vs. 13.2 for SEMAC; *p* ≤ 0.043). In soft tissue, WARP performed significantly better than the other two advanced sequences (median 207.9 vs. 74.4 for SEMAC and 51.9 for UTE; *p* ≤ 0.013; see Table 3). Moreover, CNR values, reflecting contrast between hard and soft tissues relative to background noise, were significantly higher for WARP compared to SEMAC, and both performed significantly better than UTE (medians: 184.8 for WARP, 59.6 for SEMAC, and 23.5 for UTE; *p* ≤ 0.043; see Figure 2 and Table 4).

**Table 3:** SNR values for cortical bone and muscle in SEMAC, UTE and WARP and their comparisons.

Sequence	SNR cortical bone, median (IQR)	SNR muscle, median (IQR)
UTE	30.0 (25.8–34.3)	51.9 (48.4–61.1)
SEMAC	13.2 (12.5–13.8)	74.4 (65.4–79.9)
WARP	23.8 (18.1–26.5)	207.9 (144.0–230.1)

Comparison SNR cortical bone	*p*-value (adjusted)	Comparison SNR muscle	*p*-value (adjusted)
UTE vs. SEMAC	<0.001*	UTE vs. SEMAC	0.307
UTE vs. WARP	0.199	UTE vs. WARP	<0.001*
SEMAC vs. WARP	0.043*	SEMAC vs. WARP	0.013*

Upper Table: Signal-to-noise ratio (SNR) values for SEMAC, UTE and WARP, values presented as median and interquartile range (IQR).

Lower Table: Pairwise comparisons of the three sequences. Previous Shapiro–Wilk test revealed not normally distributed data, not shown. A Bonferroni correction was applied to account for multiple comparisons and determine significance.

UTE and WARP showed significantly higher SNR values in the cortical bone compared to SEMAC. SNR values in muscle were significantly higher for WARP compared to SEMAC and UTE.

* *p* < 0.05.

SNR, signal-to-noise ratio; UTE, ultrashort echo time; SEMAC, slice encoding for metal artifact correction; WARP, vendor-specific artifact reduction techniques (Siemens Healthineers, Forchheim, Germany).

**Table 4:** CNR values for SEMAC, UTE and WARP.

Sequence	CNR, median (IQR)
UTE	23.5 (20.5–27.3)
SEMAC	59.6 (51.4–66.5)
WARP	184.8 (122.7–206.3)

Comparison CNR values	*p*-value (adjusted)
UTE vs. SEMAC	0.043*
UTE vs. WARP	<0.001*
SEMAC vs. WARP	0.043*

Upper Table: Contrast-to-noise ratio (CNR) values for SEMAC, UTE and WARP, values presented as median and interquartile range (IQR).

Lower Table: Pairwise comparisons of the three sequences. Previous Shapiro–Wilk-Test revealed not normally distributed data, not shown. A Bonferroni correction was applied to account for multiple comparisons and determine significance.

* *p* < 0.05.

CNR, contrast-to-noise ratio; UTE, ultrashort echo time; SEMAC, slice encoding for metal artifact correction; WARP, vendor-specific artifact reduction techniques (Siemens Healthineers, Forchheim, Germany).

**Figure 3:**
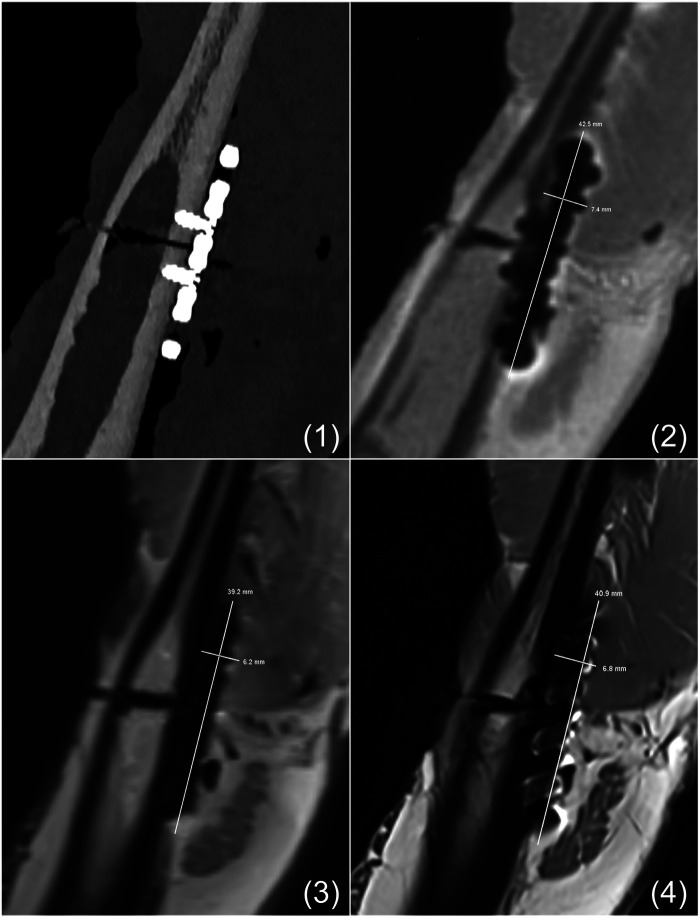
Quantification of artifact extent. Sample images for artifact quantification, from left to right and from top to bottom: (1) Gold standard photon-counting CT, (2) UTE, (3) SEMAC, and (4) WARP. In an exploratory manner, the length and width of the metallic artifacts caused by plate osteosynthesis were measured and subsequently artifact area was calculated by assuming rectangular shape. CT, computed tomography; MRI, magnetic resonance imaging; UTE, ultrashort echo time; SEMAC, slice encoding for metal artifact correction; WARP, vendor-specific artifact reduction techniques (Siemens Healthineers, Forchheim, Germany).

Two-dimensional artifact quantification revealed that SEMAC had the least artifact extent of the five investigated MRI sequences, followed by WARP, UTE, DIXON, and T1 (see Figure 3 and Table 5). Friedman test yielded significant differences in measured artifact extent between the five sequences. *Post-hoc* analysis showed that SEMAC was significantly superior to WARP (*p* = 0.004), UTE (*p* = 0.003), and DIXON, as well as T1 (*p* < 0.001). In addition, UTE and WARP each showed significantly lower artifact extent than DIXON and T1 (*p* < 0.001). Differences between UTE and WARP as well as the differences between DIXON and T1 were not significant.

**Table 5:** Quantification of artifact extent.

Modality/Sequence	Length (mean ± SD) in mm	Width (mean ± SD) in mm	Area (mean ± SD) in mm^2^	Relative artifact extent to CT (%)
CT—130 keV	20.47 ± 9.01	2.42 ± 1.14	56.07 ± 44.19	
MRI—UTE	25.98 ± 10.39	4.29 ± 1.57	123.48 ± 83.54	220%
MRI—SEMAC	22.23 ± 8.75	3.15 ± 1.06	75.75 ± 48.22	135%
MRI—WARP	24.56 ± 9.14	4.28 ± 1.70	117.29 ± 84.61	209%
MRI—DIXON	28.94 ± 10.60	5.77 ± 2.08	182.56 ± 116.81	326%
MRI—T1	31.42 ± 12.01	5.57 ± 1.76	189.78 ± 119.91	338%

Measurements of two-dimensional artifact extent determined for CT and the five MRI sequences. Quantitative artifact extent was measured in axial plane; length and width were measured, and area was calculated by assuming rectangular shape (as shown in Figure 3). Values are presented as mean ± the corresponding standard deviation (SD) in millimeters (mm) and square millimeters (mm^2^). Furthermore, relative artifact extent compared to CT was determined for the different MRI sequences based on area, presented as percentage values.

CT, computed tomography; MRI, magnetic resonance imaging; keV, kilo-electron volts; UTE, ultrashort echo time; SEMAC, slice encoding for metal artifact correction; WARP, vendor-specific artifact reduction techniques (Siemens Healthineers, Forchheim, Germany); DIXON, DIXON-based imaging; T1, T1-weighted turbo spin echo.

## 4 Discussion

This *ex vivo* study demonstrates that advanced MRI sequences significantly improve postoperative image interpretability in the presence of mandibular osteosynthesis material compared with conventional T1-TSE and DIXON imaging. UTE, WARP and SEMAC showed complementary strengths in soft-tissue visualization, osseous assessment, and artifact reduction. These findings support our hypothesis that advanced artifact-reduction sequences provide superior diagnostic interpretability and reduced artifact extent compared to conventional T1-weighted TSE and DIXON imaging.

Previous studies have demonstrated the feasibility of artifact-reduction techniques such as SEMAC and UTE *in vitro* or in non-trauma settings like in orthopedic surgery and in dental implantology (16, 23, 24). However, comparative data in a standardized postoperative mandibular fracture model remain limited. In contrast to prior work, our study systematically evaluates multiple advanced MRI sequences under controlled conditions, including both qualitative and quantitative metrics. This study provides controlled comparative data on sequence-specific performance of dedicated MRI sequences in postoperative maxillofacial imaging.

From a clinical perspective, these findings highlight the potential of MRI as a radiation-free adjunct to CT, particularly in patients requiring repeated imaging. Beyond fracture assessment, MRI has the potential to enable comprehensive evaluation of dental structures, alveolar bone, adjacent soft tissues, and neural structures within a single examination, supporting a more integrated postoperative assessment (7, 9, 21, 25–27). From a research perspective, this study provides controlled experimental data supporting sequence-specific optimization and may guide future *in vivo* studies evaluating MRI-based postoperative assessment protocols in maxillofacial trauma. The presented study extends previous *in vitro* findings by evaluating these techniques in an *ex vivo* fracture model (16).

Several limitations should be acknowledged. The *ex vivo* design does not account for motion, postoperative edema, hemorrhage, or patient-related factors, which may influence image quality in clinical practice. Second, physiological perfusion and contrast dynamics were absent. Additionally, the evaluated sequences represent vendor-specific implementations with optimized in-house parameters, which may limit generalizability. Apart from that, quantitative measurements were only performed in an exploratory manner and should be interpreted as supportive of the qualitative assessment. Furthermore, the limited sample size of this proof-of-concept *ex vivo* study precluded robust estimation of effect sizes, and therefore statistical results should be interpreted with caution. Finally, MRI remains associated with higher costs and more limited availability compared with conventional dental imaging modalities, including orthopantomography (OPG), which may restrict its routine implementation in postoperative imaging. However, MRI provides complementary three-dimensional and soft tissue information beyond the assessment achievable with OPG. *In vivo* validation is therefore required to confirm these findings and determine their impact on clinical decision-making and cost-effectiveness.

## 5 Conclusion

Advanced MRI sequences substantially improve postoperative imaging of mandibular fractures by reducing metal-related artifacts and enhancing diagnostic interpretability. A dedicated MRI protocol incorporating WARP, UTE, and SEMAC may complement CT by enabling radiation-free assessment of both osseous and soft-tissue postoperative findings. Further *in vivo* studies and clinical validation are warranted to confirm these results and assess their impact on patient outcomes.

## Data Availability

The raw data supporting the conclusions of this article will be made available by the authors, without undue reservation.
